# Supplementary material for: Urban land patterns can moderate population exposures to climate extremes over the 21st century
Source: Nat Commun. 2023 Oct 26;14:6536. doi: 10.1038/s41467-023-42084-x (PMC10603141; doi:10.1038/s41467-023-42084-x)
Supplement: Supplementary file 1 — Supplementary Information [file 41467_2023_42084_MOESM1_ESM.pdf]

Supplementary Information:

Urban Land Patterns Can Moderate Population Exposures to Climate Extremes over the 21st Century (Gao & Bukovsky)

SI Table 1. Scenarios analyzed in this research, combining different climate, population, and land use conditions at the beginning (BOC) and the end (EOC) of the 21st century, using the Shared Socioeconomic Pathways and the Representative Concentration Pathways (the SSP-RCP scenario framework).

	Scenario Names	Climate Conditions	Population Conditions	Land Use Conditions
Key Scenarios	historical	BOC	BOC	BOC
	climate	EOC (RCP 8.5)	BOC	BOC
	climate+pop	EOC (RCP 8.5)	EOC (SSP 5)	BOC
	climate+pop+landUse	EOC (RCP 8.5)	EOC (SSP 5)	EOC (SSP 5)
Helper Scenarios	climate+landUse	EOC (RCP 8.5)	BOC	EOC (SSP 5)
	pop	BOC	EOC (SSP 5)	BOC

SI Table 2. Comparison of annual population exposures to four climate extremes at the beginning (BOC) vs. the end (EOC) of the 21st century: Perspectives of both total and per capita exposures. For an example grid, if its annual per capita exposure is 30 days per person per year, and its population size is 1000, the grid's annual total exposure is 30 * 1000 = 30,000 person days.

		Total Exposure		Per Capita Exposure		
		beginning of the 21st century	end of the 21st century	beginning of the 21st century	end of the 21st century	end of the 21st century
scenario		historical	climate+pop+landUse	historical	climate (climate effects only)	climate+pop+landUse (climate & urban effects)
Hot Days	Unit	billion person days		days per person per year		
	National Total/Average	1.975	26.849	7	32	41
	in not developed areas	64%	26%	7	30	29
	in low-density development	22%	32%	8	33	42
	in mid-to-high-density development	14%	42%	8	38	53
Cold Days	Unit	billion person days		days per person per year		
	National Total/Average	15.078	16.967	54	28	26
	in not developed areas	73%	50%	57	31	35
	in low-density development	17%	26%	48	22	21
	in mid-to-high-density development	11%	24%	48	20	19
Heavy Rainfalls	Unit	million person hours		hours per thousand persons per year		
	National Total/Average	53.593	402.673	192	601	608
	in not developed areas	69%	35%	192	593	592
	in low-density development	19%	32%	192	610	633
	in mid-to-high-density development	12%	32%	192	634	602
Severe Thunderstorm Environments	Unit	billion person days		days per person per year		
	National Total/Average	30.765	76.909	110	118	116
	in not developed areas	73%	38%	116	123	122
	in low-density development	17%	32%	102	112	120
	in mid-to-high-density development	10%	30%	89	101	106

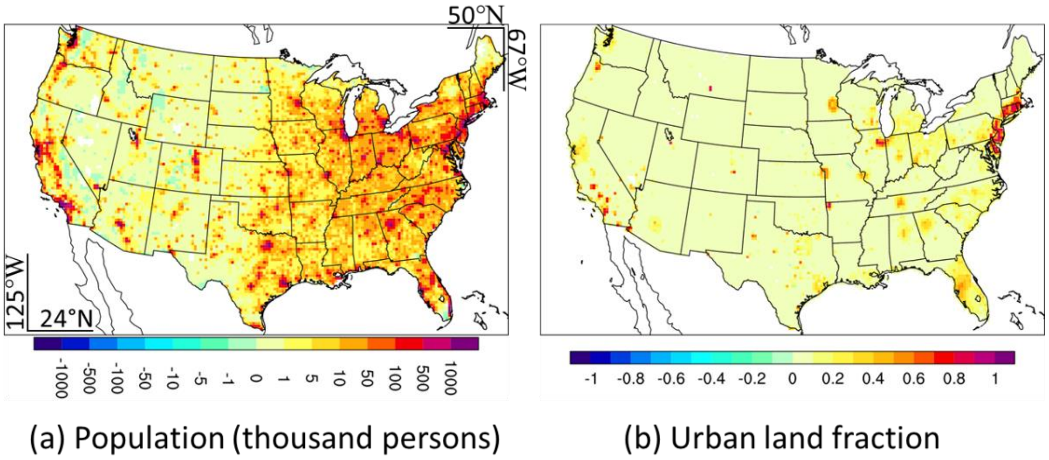

SI Figure 1. Socioeconomic projections under the Shared Socioeconomic Pathway 5 (SSP5) scenario: change over the 21st century in population and urban land.

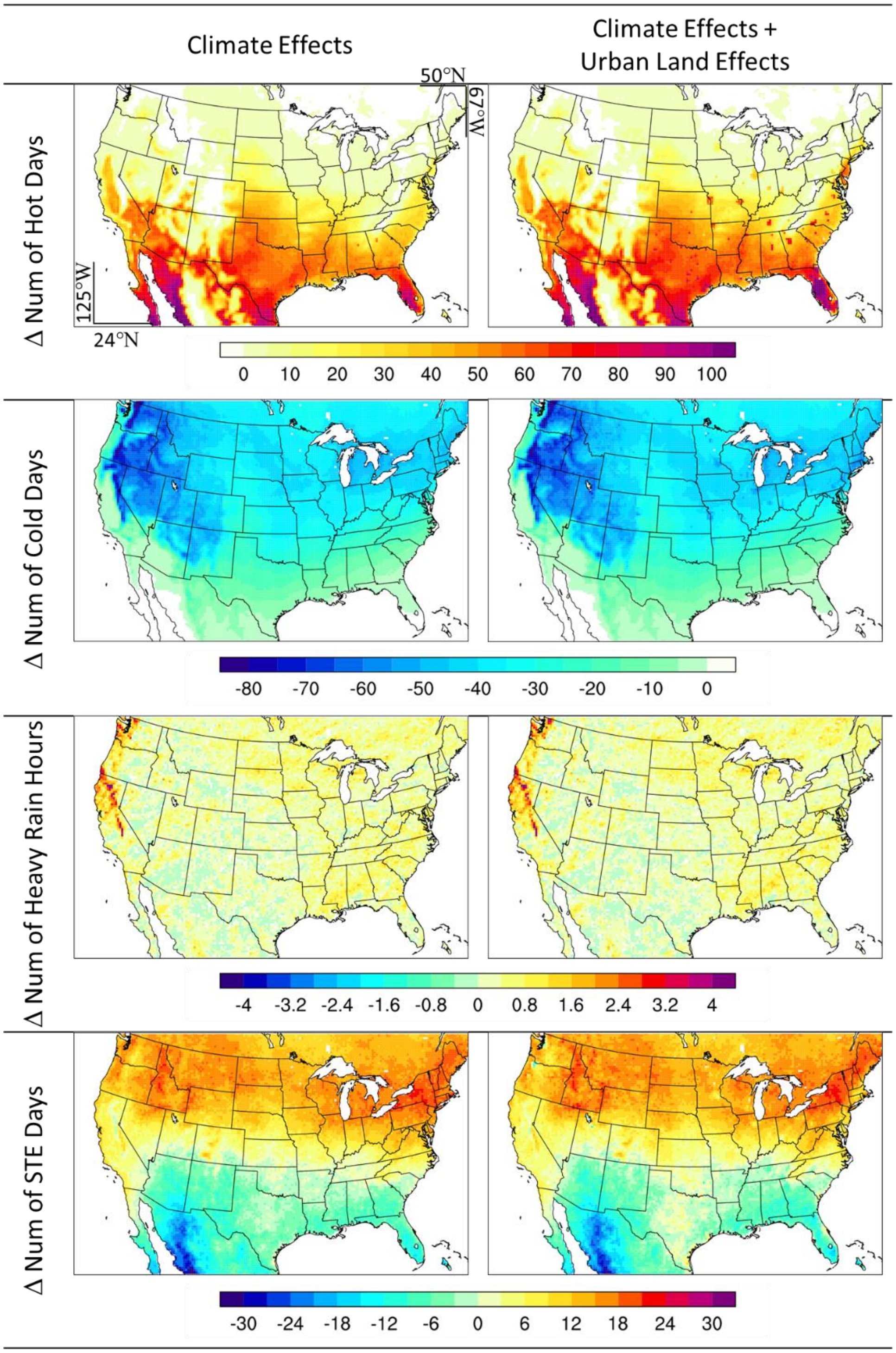

SI Figure 2. Climate projections under the Representative Concentration Pathway 8.5 (RCP8.5) scenario: change over the 21st century in four climate extremes (hot days, cold days, heavy rainfalls, and severe thunderstorm environments).

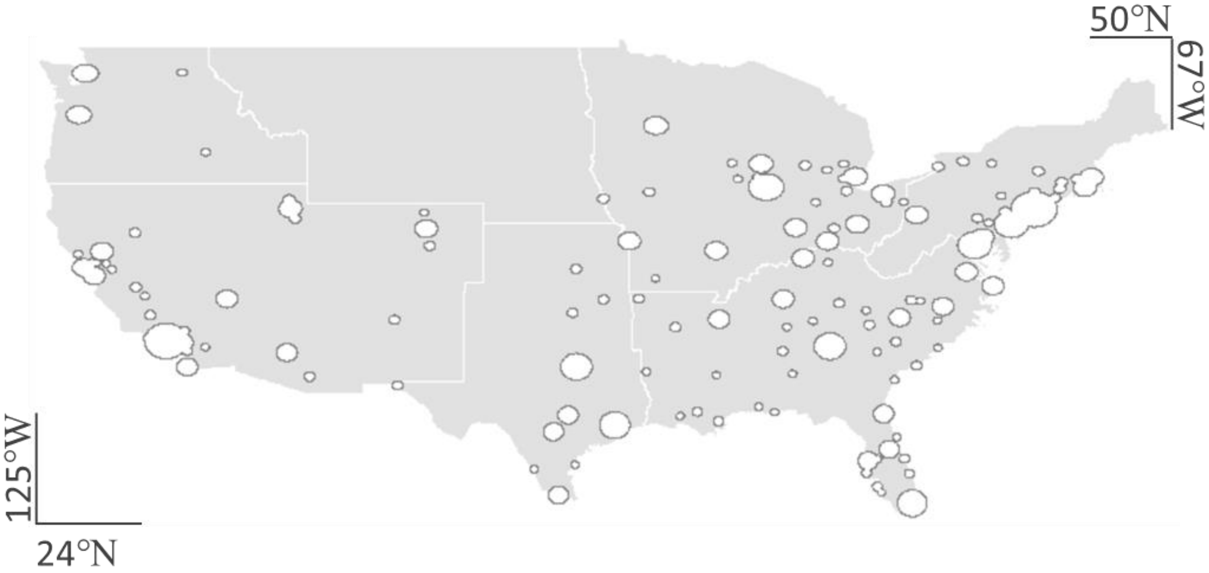

SI Figure 3. Spatial extents examined for 109 sizeable urban centers (i.e., with a population size greater than 300,000).

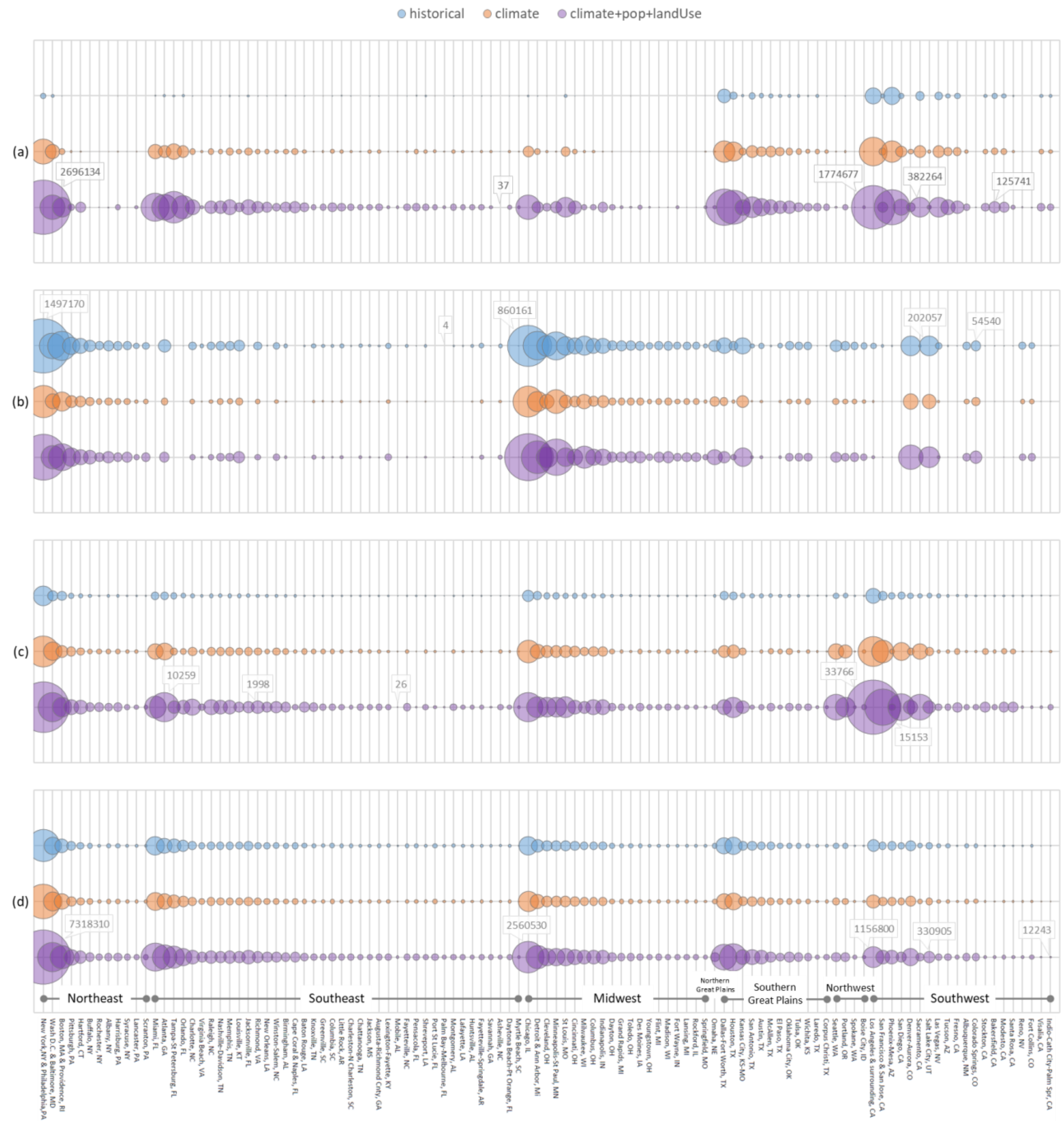

SI Figure 4. Population exposures in 109 sizeable urban centers to four climate extremes: (a) hot days, (b) cold days, (c) heavy rainfalls, and (d) severe thunderstorm environments.

The urban centers are grouped by climate regions, and within each region are ordered by descending population size from left to right. (Units: a, b, d – person days; c – person hours)

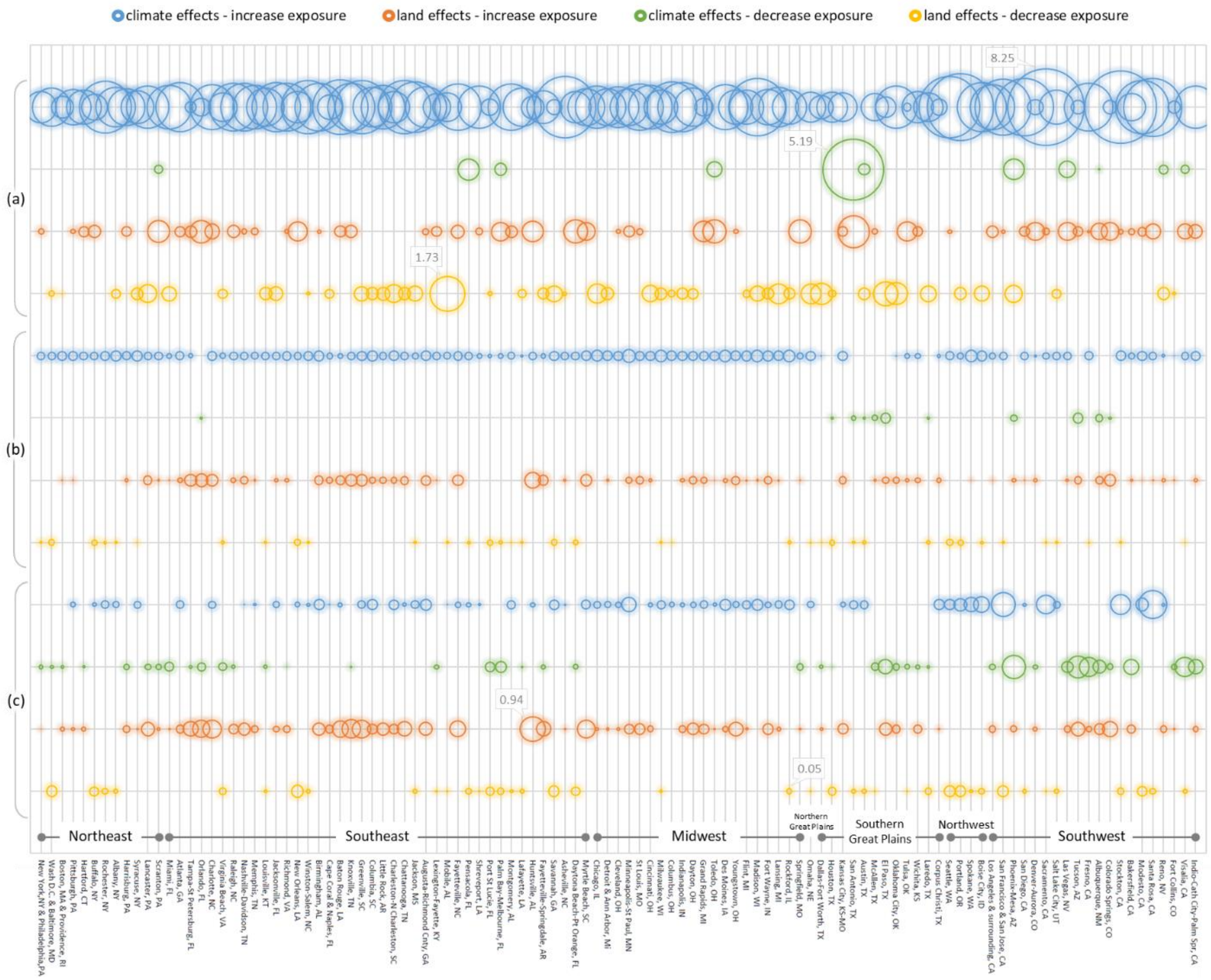

SI Figure 5. Effects of 21st-century climate change and urban land expansion in 109 sizeable urban centers on (a) population exposure to heavy rainfalls, (b) annual total precipitation, and (c) summer (Jun, Jul, Aug) total precipitation. The urban centers are grouped by climate regions, and within each region are ordered by descending population size from left to right. Overall, changes in annual total precipitation are minimal, changes in summer total precipitation are more, and changes in heavy rainfalls notably more. The U.S. total precipitation at the end of the 21st century (EOC) is about 1.1 times the beginning-of-the-century (BOC) total, while the EOC amount of heavy rainfalls is more than 3 times the BOC amount.
